# The Impact of Caring for a Child With Juvenile Idiopathic Arthritis on Caregiver Quality of Life

**DOI:** 10.1002/acr2.90156

**Published:** 2026-09-24

**Authors:** Deborah A. Marshall, Ana Claudia Fuhrmann, Rodrigo Dal Ben, Gillian R. Currie, Rae S. M. Yeung, Sebastiaan J. Vastert, Nico Wulffraat, Joost F. Swart, Susanne Benseler, the UCAN CAN‐DU and UCAN CURE Consortia, Rae Yeung, Shirley Tse, Deborah Levy, Ronald Laxer, Brian Feldman, Jennifer Ji Young Lee, Alisa Rachlis, Susanne Benseler, Nicole Johnson, Muhammed Dhalla, Paivi Miettunen, Heinrike Schmeling, Marinka Twilt, Rebeka Stevenson, Deborah Marshall, Andrea Human, David Cabral, Jaime Guzman, Kim Morishita, Kristin Houghton, Lori Tucker, Tommy Gerschman, Herman Tam, Mercedes Chan, Ross Petty, Roman Jurencak, Nadia Luca, Ciarán Duffy, Tala El Tal, Roberta Berard, Jonathan Park, Erkan Demirkaya, Lily Lim, Adam Huber, Bianca Lang, Elizabeth Stringer, Chelsea DeCoste, Suzanne Ramsey, Dax G. Rumsey, Daniah Basodan, Lillian Lim, Hon Yan Ng, Jeanine McColl, Tara McGrath, Michelle Batthish, Tania Cellucci, Liane Heale, Claire LeBlanc, Gaëlle Chédeville, Rosie Scuccimarri, Sarah Campillo, Piya Lahiry, Paul Dancey, Nickolas Blanchette, Alan Rosenberg, Mehul Jariwala, Tristan Kerr, Kate Neufeld, Clare Hutchinson, Gordon Soon, Dieneke Schonenberg, Mariken Gruppen, Merlijn van den Berg, Giske Biesbroek, Philomine van Pelt, Marleen Verkaaik, Sylvia Kamphuis, Danielle Brinkman, Petra Hissink Muller, Ellen Schatorje, Esther Hoppenreijs, Annet van Royen‐Kerkhof, Sebastiaan Vastert, Berent Prakken, Erika Van Nieuwenhove, Joost Swart, Marc Jansen, Nico Wulffraat, Elizabeth Legger, Wineke Armbrust, Joseph Cafazzo, Maarten IJzerman, Alexander Mosoiu, Amy Xu, Arthur Cheng, Bruno Pereira, Trang Duong, Harper Cheng, Brenleigh Jebb, Gillian R. Currie, Rodrigo Dal Ben, Ravneet Sran, Elodie Boudes, Michelle Kip, Sytze de Rook, Regina de Geus

**Affiliations:** ^1^ Department of Community Health Sciences, Cumming School of Medicine University of Calgary Calgary Alberta Canada; ^2^ Alberta Children's Hospital Research Institute University of Calgary Calgary Alberta Canada; ^3^ Department of Medicine, Cumming School of Medicine University of Calgary Calgary Alberta Canada; ^4^ Department of Pediatrics, Cumming School of Medicine University of Calgary Calgary Alberta Canada; ^5^ Department of Paediatrics, Immunology, and Medical Science The Hospital for Sick Children and University of Toronto Toronto Ontario Canada; ^6^ Department of Pediatric Immunology and Rheumatology Wilhelmina Children's Hospital, University Medical Center Utrecht Utrecht the Netherlands; ^7^ Faculty of Medicine University Medical Center Utrecht Utrecht the Netherlands; ^8^ Children's Health Ireland Dublin Ireland

## Abstract

**Objective:**

Juvenile idiopathic arthritis (JIA) impacts children and their families, often increasing caregiver burden and diminishing quality of life. This study assessed the care‐related quality of life of parent‐caregivers of children with JIA using the Care‐Related Quality of Life instrument (CarerQol), identified the most affected caregiving dimensions, and explored associations with sociodemographic and clinical factors.

**Methods:**

We analyzed baseline data from the Canada‐Netherlands Personalized Medicine Network in Childhood Arthritis and Rheumatic Diseases (UCAN CAN‐DU) study. Clinical data including symptoms, disease activity, and caregiver‐reported data were collected. Caregivers of children under 18 completed the CarerQol, a preference‐weighted caregiver quality‐of‐life instrument from which a utility score (0–100, worst to better caregiving situation) can be calculated, as well as a well‐being visual analog scale (VAS; 0–10, completely unhappy to happy). Stepwise linear regression examined associations between CarerQol utility and VAS; and sociodemographic and clinical variables, with model selection based on goodness of fit.

**Results:**

Among 439 caregivers, 86% were mothers, the mean age was 41 years, and 44% of children had oligoarticular JIA. The mean CarerQol scores were utility of 81.5 (SD 10.8); VAS of 7.3 (SD 1.7). Most caregivers reported fulfillment and caregiving support. Over 40% reported problems in physical health, mental health, and combining caregiving with daily activities. Lower utilities were associated with receiving extra help, having physical health conditions, caring for children using injectable medication, and caring for male children. Higher utilities were associated with living with a partner, caring for children aged 10 to 15, and residing in the Netherlands (*P* < 0.05).

**Conclusion:**

Many caregivers experience substantial burden, particularly in physical and mental health, and managing caregiving alongside daily responsibilities. Child‐ and family‐related factors are key variables associated with caregiving quality of life. Results emphasize the need for tailored interventions and support systems to improve JIA caregivers’ well‐being.

## INTRODUCTION

Juvenile idiopathic arthritis (JIA) is the leading cause of musculoskeletal pain and disability in children, often associated with lifelong physical, emotional, and social challenges.^1^ JIA is a term for heterogeneous chronic arthritis conditions of unknown cause that affect children aged 16 or younger and persist for at least six weeks.^2^ In 2017, more than two million children worldwide were living with JIA.^3^

**SIGNIFICANCE & INNOVATIONS**

- This is the first comprehensive study of the Care‐Related Quality of Life instrument (CarerQol) among caregivers of children with juvenile idiopathic arthritis.
- Caregivers reported major challenges with physical health, mental health, and combining care with daily responsibilities—dimensions overlooked in previous studies.
- Lower care‐related quality of life was associated with poorer caregiver and partner health, higher care demands (eg, children on injectable medications, with extra‐articular symptoms or with comorbidities), and unmet support needs.
- This study provides utility‐based caregiver outcomes to support health economic evaluations and policy decisions in pediatric rheumatology.
- Identifies higher‐risk caregiver subgroups, guiding more tailored and equitable support strategies.

The symptoms and management of JIA significantly impact the lives of both children and their families.^4^, ^5^, ^6^, ^7^ Caregivers often face numerous challenges, including monitoring symptoms, managing pain and discomfort, handling medication side effects, addressing children's behavioral issues, and managing financial strain due to frequent outpatient visits and hospitalizations.^4^, ^5^, ^6^, ^7^ The unpredictable nature of JIA further raises concerns about the children's present and future well‐being^6^ which contributes to increasing caregiving burden and reducing quality of life.

These valuable insights into the complex emotional, social, and practical demands of caregiving primarily come from qualitative studies.^4^, ^5^, ^6^ Few quantitative studies have explored key aspects of the caregiving experience, such as burden,^8^ mental health,^9^ stress,^10^ and quality of life,^11^, ^12^ in the context of JIA. Moreover, most of these studies have relied on generic instruments not specifically designed to measure care‐related impacts, potentially overlooking unique and multifaceted challenges caregivers face. Even tools focused on the caregiving experience frequently do not enable the estimation of utility scores, which limits the ability to quantify the impact of JIA on caregivers to inform health economic evaluations, resource allocation, and policy decisions in pediatric rheumatology.

Measuring care‐related quality of life among caregivers of children with JIA and identifying related factors provides critical insights into the multidimensional impact of caregiving and the diverse experiences within caregiver populations. Identifying patterns of vulnerability and resilience can support more informed service design and delivery, as well as guide targeted interventions and strategies that address the most unmet needs to reduce caregivers’ burden and enhance their well‐being.

The Care‐Related Quality of Life instrument (CarerQol) is a validated tool to comprehensively assess and quantify the impact of informal caregiving, capturing both the positive and negative aspects of providing care. It integrates caregivers’ self‐report experiences alongside a well‐being evaluation, enabling the generation of utility scores.^13^ The CarerQol instrument has been used with caregivers of children with various health conditions, including autism spectrum disorder (ASD), cystic fibrosis, and neuromuscular disorders.^14^, ^15^, ^16^, ^17^, ^18^, ^19^, ^20^, ^21^, ^22^, ^23^, ^24^, ^25^, ^26^ However, its use in the context of JIA remains limited, with only one earlier study from our research team reporting preliminary results.^27^ That study provided initial insights and found higher CarerQol scores (higher care‐related quality of life) among caregivers living in the Netherlands, who were employed, and with better health‐related quality of life. In comparison, lower CarerQol scores (lower care‐related quality of life) were associated with receiving additional help with childcare.

The current study builds upon that preliminary work by using a larger sample size to provide a more comprehensive assessment of the care‐related quality of life of caregivers of children with JIA. It aims to quantify the care‐related quality of life measured by the CarerQol instrument, identify the caregiving dimensions most often affected, and explore the association of the care‐related quality of life with sociodemographic and clinical factors.

## MATERIALS AND METHODS

We analyzed baseline data collected by the Canada‐Netherlands Personalized Medicine Network in Childhood Arthritis and Rheumatic Diseases & Precision Decisions for Childhood Arthritis (UCAN CAN‐DU and UCAN CURE) study between September 7, 2019, and June 16, 2025.^27^, ^28^, ^29^ UCAN CAN‐DU and UCAN CURE is a prospective, multicenter study conducted in all pediatric rheumatology clinics across Canada and the Netherlands, which included consecutive children under 18 years of age with arthritis at defined stages of disease (newly diagnosed, initiating or discontinuing a biologic treatment), and their caregivers (see Appendix A for a list of UCAN CAN‐DU and UCAN CURE Consortia members). Families of children with JIA attending rheumatology clinics were invited to participate in the study. Parents provided informed consent for them and their child's participation, and patients assented. In the Netherlands, children over 16 could provide their own consent. In Canada, if a participant under 18 when initially enrolled subsequently turned 18, consent as an adult was additionally collected, and those who were younger and had decision‐making capability could provide their own consent. Ethics approval was obtained from the Conjoint Health Research Ethics Board at the University of Calgary (Canada) (REB17–1563), University of Toronto (Canada) (CTO #1771), and the Ethical Board of Utrecht (the Netherlands) (18–474). Patient and public involvement in the study was facilitated by the Patient Engagement Advisory Committee.

### Measurements

Data were prospectively captured in the UCAN eHealth platform, a secure, multilingual, web‐based app integrating clinical, treatment, and patient‐ and caregiver‐reported information. Clinical research staff entered children's demographic, clinical, and treatment data, including sex, date of birth, comorbidities, musculoskeletal manifestations (joint pain, muscle pain, and morning stiffness), extra‐articular manifestations (eg, nail pitting, dactylitis, uveitis, and rash), JIA subtype (eg, oligoarticular, enthesitis‐related arthritis, and systemic), clinical Juvenile Arthritis Disease Activity Score 10 (cJADAS10), disease status, and medication use (drug, dose, and route of administration). JIA activity states were defined according to JIA subtype and cJADAS scores as follows: for oligoarthritis, inactive disease (≤1.1), minimal disease activity (1.2–4), moderate disease activity (4.1–12), and high disease activity (>12); for polyarthritis, inactive disease (≤2.5), minimal disease activity (2.6–5), moderate disease activity (5.1–16), and high disease activity (>16).^30^ Missing cJADAS10 scores were estimated using linear prediction based on provider global assessment scores, which were strongly correlated with cJADAS10 (Pearson's ρ = 0.86, *P* < 0.001).

At baseline, caregivers provided information on sociodemographic, help received to care for the children with JIA, and CarerQol responses. Sociodemographic characteristics included country of residence, gender, age, relationship with the child, living with a partner, number of dependent children other than the child with JIA, education level, employment status, presence of physical and mental health conditions, household income, partners’ employment status, and partners’ physical and mental health conditions. Our analyses included only families with complete CarerQol, sociodemographic and clinical data at baseline. The CarerQol instrument consists of two parts: the CarerQol‐7D and the CarerQol visual analog scale (VAS). The first contains seven dimensions that describe the caregiving situation: fulfillment in caring, relational problems with the person receiving care, mental health problems, issues combining care with daily activities, financial problems due to caring, support for caregiving, and physical health issues.^13^ Fulfillment and support represent positive experiences, whereas the other dimensions reflect negative experiences. The VAS is a happiness measure to assess the effect of caregiving on the caregiver's well‐being.^13^

For each domain, caregivers rate their experience as “no” (scored as 0), “some” (scored as 1), or “a lot of” (scored as 2). The combination of answers for each caregiver represents a care status, to which an equivalent utility score can be assigned based on discrete choice experiments from the general population.^31^ The CarerQol allows 2,187 (7^3^) distinct care statuses^13^ and the utility score ranges from 0, worst caregiving situation, to 100, best caregiving situation.^25^ For countries without utility scores, the level sum scores are used. The level sum score ranges from 0, the best caregiving situation, to 14, the worst caregiving situation. For the VAS, caregivers rate their overall well‐being on a discrete scale from 0 to 10, where 0 means completely unhappy and 10 means completely happy.^13^ The CarerQol questions assess the caregiving situation and its impact at the time caregivers complete the questionnaire.

### Statistical analysis

We report descriptive sociodemographic characteristics of caregivers and children, as well as relevant clinical information, by summarizing continuous measures as proportions, means, and SDs. For measures with nonnormal distributions (assessed with the Jarque‐Bera normality test^32^), we report median and interquartile ranges (IQRs). Additionally, we present the CarerQol‐7D utility score, level distribution by domain, along with the CarerQol‐VAS score. For the CarerQol‐7D utility score, we adopted the utility scores for the Dutch population,^31^ given the absence of Canadian utility scores. Level sum scores are also reported to support comparability with studies that do not use utility scores. In addition, we present “extra help to care,” which is derived from an additional study‐specific question rather than the CarerQol instrument.

Moreover, we constructed two separate exploratory linear regression models, one with the CarerQol‐7D utility score and one with the CarerQol‐VAS score as the dependent variable. Independent variables (ie, multivariable covariates) were selected based on existing literature^17^, ^18^, ^21^, ^22^, ^25^ and included characteristics related to caregivers, children, and clinical factors. Caregivers’ sociodemographic variables included the following: country of residence (Canada or the Netherlands), gender, age, relationship with the child, living with a partner, number of dependent children other than the child with JIA, education level, employment status, presence of physical and mental health conditions, household income, and extra help received to care for their children with JIA. Children's characteristics and clinical variables included: sex, age, presence of other health conditions, musculoskeletal and extra‐articular signs and symptoms, JIA classification, disease activity, disease status, and use of injectable medication. To further explore the impact of caregivers’ partners, we also ran additional models with the same variables and including the partners’ employment status and physical and mental health conditions. These models were restricted to the subset of caregivers who provided this information.

Outliers on both outcome measures were evaluated.^33^ Outliers were kept in the utility score model, as they remained within a plausible range and did not distort the overall distribution. In contrast, outliers in the VAS score model (n = 13) were excluded as their distribution substantially differed from the overall VAS distribution, which could compromise the validity of the analysis. The selection of models involved a two‐step process. First, all variables were included. Second, a forward and backward stepwise selection was applied, guided by Akaike information criteria^34^ to determine the best‐fitting model for each outcome. The final models included nine covariates. Robust linear regressions were also performed as a sensitivity analysis to reduce the influence of outliers via M‐estimation, with inference based on heteroskedasticity‐robust (sandwich) SEs. All statistical analyses were conducted in R,^33^ with a significance set at an α of 0.05.

### Data availability

Data are available on reasonable request and according to the UCAN CAN‐DU and UCAN CURE data sharing policy.

## RESULTS

### Sample description

Of the 1,432 families included in the UCAN CAN‐DU and UCAN CURE study, 496 had CarerQol baseline data. After excluding those with unconfirmed JIA diagnosis and incomplete baseline demographic and clinical data, 439 families were included in this analysis (Figure S1). Most of the caregivers were mothers (85.9%), living with a partner (87.7%), with a median age of 41.42 years (IQR 6.9). Employment status showed that 86.4% of the caregivers and 84.3% of their partners had a paid occupation. Over 40% of the caregivers reported having physical and/or mental health conditions, and 23.4% of their partners also reported physical and/or mental health conditions, as presented in Table 1.

**Table 1:** Caregivers’ and spouse or partners’ sociodemographic characteristics*

Variable	Overall	Canada	The Netherlands
Number of respondents	439	201	238
Sex, n (%)			
Female	377 (85.7)	175 (87.1)	202 (84.9)
Male	62 (14.1)	26 (12.9)	36 (15.1)
Age, mean (SD), y	41.4 (6.9)	41.3 (6.8)	41.6 (7.0)
Relationship with the child, n (%)			
Mother	378 (85.9)	175 (87.1)	203 (85.3)
Father	61 (13.9)	26 (12.9)	35 (14.7)
Living with a spouse or partner, n (%)			
Yes	386 (87.7)	176 (87.6)	210 (88.2)
No	53 (12.0)	25 (12.4)	28 (11.8)
Number of dependent children, other than patient with JIA, median (IQR)	1.0 (1.0–2.0)	1.0 (1.0–2.0)	1.0 (1.0–2.0)
Highest level of education, n (%)			
University	269 (61.3)	129 (64.2)	140 (58.8)
Technical trade school	123 (28.0)	43 (21.4)	80 (33.6)
High school or grade school	47 (10.7)	29 (14.4)	18 (7.6)
Job situation, n (%)			
Paid	381 (86.6)	18 (83.6)	213 (89.5)
Unpaid	58 (13.2)	33 (16.4)	2 (10.5)
Job situation: spouse or partner, n (%)			
Paid	372 (84.7)	18 (83.6)	24 (85.7)
Unpaid	14 (3.2)	8 (4.0)	6 (2.5)
Not applicable	53 (12.1)	25 (12.4)	28 (11.8)
Health conditions, n (%)			
Physical health	140 (36.1)	50 (30.9)	90 (39.8)
Mental health	19 (4.9)	16 (9.9)	< 5
No condition	229 (59.0)	96 (59.3)	133 (58.8)
Health conditions: spouse or partner, n (%)			
Physical health	85 (20.4)	40 (22.0)	45 (19.2)
Mental health	12 (2.9)	10 (5.5)	< 5
No condition	266 (63.9)	107 (58.8)	159 (67.9)
Not applicable	53 (12.7)	25 (13.7)	28 (12.0)
Household income, median (IQR)^a^	43,597.0 (37,547.5–63,617.0)	65,377.0 (58,333.0–76,677.0)	37,847.5 (35,408.0–41,163.2)

* Caregivers’ characteristics, unless otherwise noted. Missing data were ignored when calculating median (IQR). IQR, interquartile range; JIA, juvenile idiopathic arthritis.

^a^ Average annual disposable household income (Source: Canada Census 2021, Netherlands Census 2020) adjusted for inflation to 2022 and converted to euro.

Table 2 presents the children's demographic and clinical characteristics. Most children were female (63.4%), with a median age of 11 years (IQR 6–15). Musculoskeletal signs and symptoms were present in 55.2%, and 12.7% of children had extra‐articular signs and symptoms. The most prevalent type of JIA was oligoarticular (44%), and moderate disease activity and high disease activity were observed in 54% and 24.4% of children with JIA, respectively.

**Table 2:** Children's demographic and clinical characteristics*

Variable	Overall	Canada	The Netherlands
Number of respondents	439	201	238
Sex, n (%)			
Male	160 (36.4)	73 (36.3)	87 (36.6)
Female	279 (63.4)	128 (63.7)	151 (63.4)
Age, median (IQR), y	11.0 (6.0–15.0)	11.0 (6.0–15.0)	11.0 (6.0–15.0)
Other health conditions, n (%)			
Yes	96 (21.8)	44 (21.9)	52 (21.8)
No	343 (78.0)	157 (78.1)	186 (78.2)
Musculoskeletal signs and symptoms, n (%)			
Yes	243 (55.2)	135 (67.2)	108 (45.4)
No	196 (44.5)	66 (32.8)	130 (54.6)
Extra‐articular signs and symptoms, n (%)			
Yes	56 (12.7)	35 (17.4)	21 (8.8)
No	383 (87.0)	166 (82.6)	217 (91.2)
Injection medication, n (%)			
Yes	267 (60.7)	112 (55.7)	155 (65.1)
No	172 (39.1)	89 (44.3)	83 (34.9)
JIA activity state,^a^ n (%)			
Inactive	52 (11.8)	27 (13.4)	25 (10.5)
Minimal	43 (9.8)	25 (12.4)	18 (7.6)
Moderate	237 (54.0)	108 (53.7)	129 (54.2)
High	107 (24.4)	41 (20.4)	66 (27.7)
JIA subtype, n (%)			
Oligoarticular^b^	193 (44.0)	69 (34.3)	124 (52.1)
Polyarticular RF‐negative	100 (22.8)	51 (25.4)	49 (20.6)
Enthesitis‐related arthritis	46 (10.5)	24 (11.9)	22 (9.2)
Systemic	37 (8.4)	19 (9.5)	18 (7.6)
Polyarticular RF‐positive	22 (5.0)	10 (5.0)	12 (5.0)
Psoriatic arthritis	19 (4.3)	16 (8.0)	< 5
Undifferentiated	22 (5.0)	12 (6.0)	10 (4.2)
Medication route, n (%)			
Multiple routes	287 (68.0)	118 (61.1)	169 (73.8)
Oral	118 (28.0)	66 (34.2)	52 (22.7)
Subcutaneous	17 (4.0)	9 (4.7)	8 (3.5)

* Missing data were ignored when calculating median (IQR). IQR, interquartile range; JIA, juvenile idiopathic arthritis; RF, rheumatoid factor.

^a^ JIA activity states were defined according to JIA subtype and clinical Juvenile Arthritis Disease Activity Score 10 (cJADAS10): for oligoarthritis, inactive disease (≤1.1), minimal disease activity (1.2–4), moderate disease activity (4.1–12), and high disease activity (>12); for polyarthritis, inactive disease (≤2.5), minimal disease activity (2.6–5), moderate disease activity (5.1–16), and high disease activity (>16).^30^ Missing cJADAS10 scores were estimated using linear prediction based on provider global assessment scores, which were strongly correlated with cJADAS10 (Pearson's ρ = 0.86, *P* < 0.001).

^b^ Oligoarticular (if <6 months), n = 119; persistent oligoarticular JIA, n = 52; extended oligoarticular JIA, n = 22.

### Care‐related quality of life

**Figure 1:**
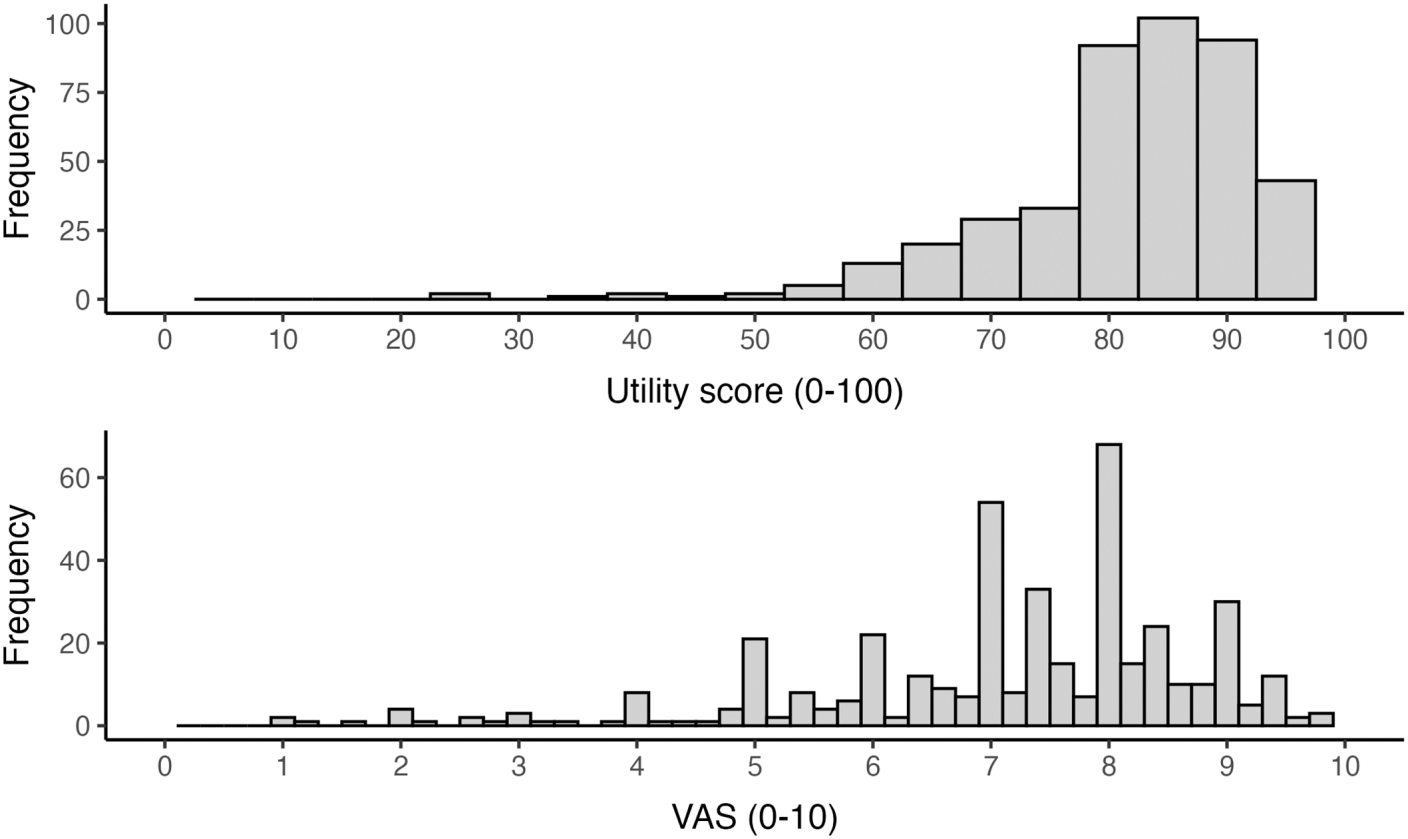
Overall Care‐Related Quality of Life instrument (CarerQol‐7D) utility and CarerQol‐VAS scores at baseline. Utility score ranges from 0 (the worst caregiving status) to 100 (the best caregiving status). VAS ranges from 0 (completely unhappy) to 10 (completely happy). VAS, visual analog scale.

Figure 1 illustrates the distribution of the CarerQol‐7D utility and CarerQol‐VAS scores. Overall, both distributions were left‐skewed. The mean utility score was 81.5 (SD 10.8), and the median was 83.9 (IQR 77.8–88.2). The mean VAS was 7.3 (SD 1.7), with a median of 7.5 (IQR 6.55–8.4). CarerQol utility and VAS scores were comparable between Canada and the Netherlands, with similar distributions observed across both countries. Level sum scores can be found in Figure S2.

**Figure 2:**
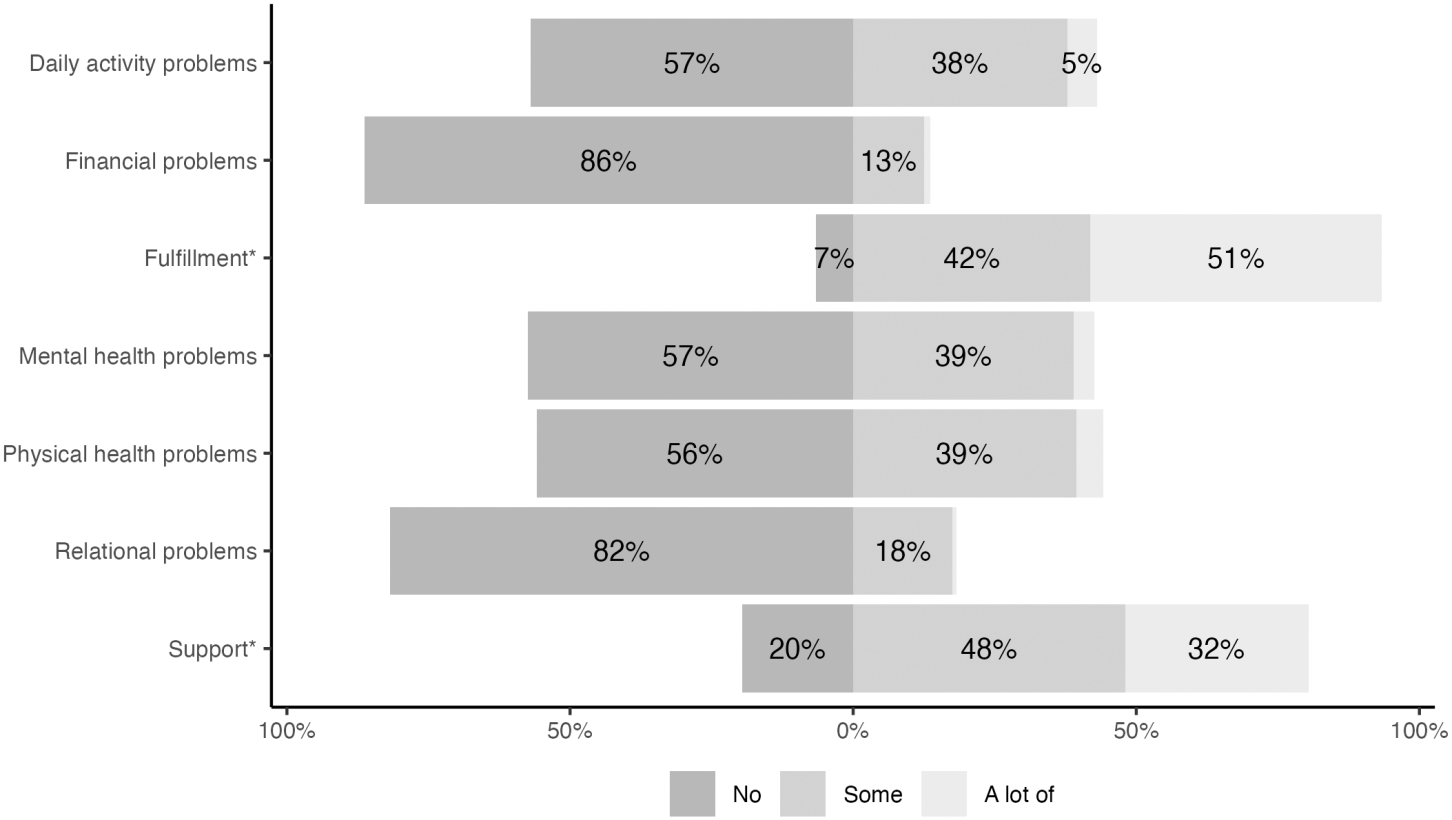
Distribution of caregivers’ responses by Care‐Related Quality of Life instrument 7D dimensions. Numbers are percentages indicating the proportion of each response. *Positive dimensions.

Figure 2 provides a detailed breakdown of the CarerQol‐7D results across the seven dimensions. Among the negative dimensions, physical health was the most frequently affected, with 44% of caregivers reporting “some” or “a lot of problems,” followed by mental health (43%), and challenges combining care with daily activities (43%). Financial (14%) and relational problems (18%) were the least often reported. Conversely, 93% of caregivers reported experiencing “some” or “a lot of” fulfillment from their caregiving experience, and 80% reported feeling supported.

### Extra help to care

Only 22% of caregivers reported receiving help from family members, friends, and/or neighbors in the three months before joining the study, and only 4% reported receiving paid help from a babysitter or house cleaner. Moreover, only 64% of caregivers reported they received the help they needed in the previous three months (Figure S3; Table S1).

### Factors associated with care‐related quality of life

Exploratory regression analyses identified significant differences across demographics and clinical variables for CarerQol‐7D utility scores and CarerQol‐VAS scores. Results for the full regression models before stepwise variable selection are presented in Table S2. Variables retained in the final stepwise models are shown in Tables 3 and 4.

**Table 3:** Stepwise regression for CarerQol‐7D utility scores*

	Only caregiver information^a^			With spouse or partner information^b^		
Predictors	Estimates	CI	*P*	Estimates	CI	*P*
(Intercept)	79.06	75.06 to 83.06	**<0.001**	84.02	81.02 to 87.01	**<0.001**
Receiving an injectable medicine	−2.56	−4.56 to −0.56	**0.012**	−2.24	−4.39 to −0.09	**0.041**
Country, the Netherlands	3.06	1.11 to 5.00	**0.002**	1.84	−0.26 to 3.94	0.085
Child sex, male	−2.16	−4.21 to −0.11	**0.039**	−2.47	−4.63 to −0.32	**0.025**
Child age group, 5–9 y	1.65	−1.43 to 4.74	0.292	2.00	−1.24 to 5.23	0.226
Child age group, 10–15 y	3.45	0.70 to 6.20	**0.014**	2.89	0.04 to 5.73	**0.047**
Child age group, 16–18 y	3.30	−0.05 to 6.65	0.054	3.59	0.05 to 7.14	**0.047**
Received extra help	−3.90	−6.24 to −1.56	**0.001**	−3.09	−5.60 to −0.58	**0.016**
Physical health condition: caregiver	−3.62	−5.58 to −1.67	**<0.001**	−2.93	−5.03 to −0.83	**0.006**
Living with a partner	3.78	0.78 to 6.78	**0.014**	–	–	–
Job situation, unpaid: spouse or partner	–	–	–	−0.83	−6.50 to 4.85	0.774
Physical health condition: spouse or partner	–	–	–	−2.15	−4.53 to 0.23	0.076
Mental health condition: spouse or partner	–	–	–	−11.31	−17.34 to −5.28	**<0.001**

* Bold values indicate results that were statistically significant (*p* < 0.05). CarerQol, Care‐Related Quality of Life instrument; CI, confidence interval.

^a^ *F*_9,429_ = 6.48, *P* ≤ 0.001; observations: 439; R^2^/R^2^ adjusted: 0.120/0.101.

^b^ *F*_11,374_ = 5.24, *P* ≤ 0.001; observations: 386; R^2^/R^2^ adjusted: 0.133/0.108.

**Table 4:** Stepwise regression for CarerQol‐VAS scores*

	Only caregiver information^a^			With spouse/partner information^b^		
Predictors	Estimates	CI	*P*	Estimates	CI	*P*
(Intercept)	7.87	7.46 to 8.28	**<0.001**	7.84	7.31 to 8.3	**<0.001**
Country, the Netherlands	−0.20	−0.47 to 0.07	0.141	−0.22	−0.57 to 0.13	0.212
Child comorbidities	−0.32	−0.64 to −0.01	**0.045**	−0.22	−0.64 to 0.19	0.294
Musculoskeletal signs and symptoms	−0.26	−0.55 to 0.02	0.068	−0.28	−0.64 to 0.08	0.123
Extra‐articular signs and symptoms	−0.60	−1.00 to −0.21	**0.003**	−0.45	−0.94 to 0.04	0.070
JIA activity state, inactive	0.91	0.42 to 1.40	**<0.001**	0.75	0.15 to 1.35	**0.015**
JIA activity state, minimal	0.39	−0.11 to 0.89	0.125	0.04	−0.56 to 0.64	0.897
JIA activity state, moderate	0.43	0.12 to 0.75	**0.007**	0.37	−0.03 to 0.77	0.069
Received extra help	−0.61	−0.91 to −0.31	**<0.001**	−0.39	−0.77 to −0.01	**0.047**
Physical health condition: caregiver	−0.50	−0.76 to −0.24	**<0.001**	−0.38	−0.71 to −0.05	**0.025**
Job situation, unpaid: spouse or partner	–	–	–	−0.20	−1.09 to 0.69	0.652
Physical health condition: spouse or partner	–	–	–	−0.32	−0.69 to 0.05	0.092
Mental health condition: spouse or partner	–	–	–	−1.80	−2.77 to −0.84	**<0.001**

* Bold values indicate results that were statistically significant (*p* < 0.05). CarerQol, Care‐Related Quality of Life instrument; CI, confidence interval; JIA, juvenile idiopathic arthritis; VAS, visual analog scale.

^a^ *F*_9,407_ = 8.97, *P* < 0.001; observations: 417; R^2^/R^2^ adjusted: 0.166/0.147.

^b^ *F*_12,373_ = 4.43, *P* ≤ 0.001; observations: 386; R^2^/R^2^ adjusted: 0.125/0.097.

For CarerQol utility scores, significantly lower utility scores were observed among caregivers who received extra help in the prior three months to care for their child with JIA (β = −3.90, *P* = 0.001), caregivers with physical health conditions (β = −3.62, *P* < 0.001), and those whose children were receiving injectable medication (β = −2.56, *P* < 0.005). Moreover, caregivers of male children had slightly lower utility scores (β = −2.16, *P* < 0.045). Conversely, significantly higher utility scores were reported by caregivers living with a spouse or partner (β = 3.78, *P* < 0.005), caregiver of children aged 10 to 15 years (β = 3.45, *P* < 0.005), and those living in the Netherlands (β = 3.06, *P* < 0.005).

For CarerQol‐VAS, lower VAS scores were significantly associated with caregivers of children who had extra‐articular signs and symptoms (β = −0.60, *P* = 0.003), caregivers with physical health conditions (β = −0.50, *P* < 0.001), those who received extra help (β = −0.461, *P* < 0.001), and caregivers of children with additional health conditions besides JIA (β = −0.32, *P* = 0.045). In contrast, caregivers of children with inactive (β = 0.91, *P* < 0.001) and moderate (β = 0.43, *P* = 0.007) disease activity reported higher VAS scores. In models incorporating characteristics of the caregiver's partner, lower utility scores were significantly associated with having a partner with a mental health condition (β = −11.31, *P* < 0.001), receiving extra help (β = −3.09, *P* = 0.001), and having a physical health condition (β = −2.93, *P* = 0.006). Caregivers of male children (β = −2.47, *P* = 0.002) and of children using injectable medication (β = −2.24, *P* = 0.004) also had lower scores. On the other hand, caregivers of older children had higher utility scores (ages 10–15 years: β = 2.89, *P* = 0.047; ages 16–18 years: β = 3.59, *P* = 0.047).

In the CarerQol‐VAS model that included partner variables, lower scores were significantly associated with caregivers whose partners had mental health conditions (β = −1.80, *P* < 0.001), caregiver who received extra help (β = −0.39, *P* = 0.047), and caregivers who had physical health conditions (β = −0.38, *P* = 0.025). On the other hand, higher VAS scores were reported by caregivers of children with inactive (β = 0.75, *P* = 0.015) disease activity.

### Reliability analyses

Robust regression analyses using the same set of predictors confirmed the significance and direction of the key associations identified in the regular regression models (Tables S3 and S4).

## DISCUSSION

This study provides the first comprehensive assessment of the care‐related quality of life among caregivers of children with JIA using the CarerQol instrument, revealing the multidimensional impact of caregiving on parental quality of life. Despite relatively high average CarerQol utility and VAS scores, dimension‐level findings indicate a mixed pattern of caregiver experiences, with many caregivers experiencing substantial burden, particularly related to physical health, mental health, and managing caregiving with daily responsibilities. These dimensions emerged as consistent areas of unmet need.

CarerQol scores in our sample are comparable to those found in studies with caregivers of children with other chronic, incurable conditions, such as ASD,^14^, ^16^ cystic fibrosis,^18^, ^19^ and drug‐resistant epilepsy.^17^ However, they were higher than those observed in more debilitating conditions, such as neuromuscular or rare genetic disorders.^20^, ^23^, ^24^ The fluctuating disease course of JIA, with periods of remission as well as nontrivial mortality risks, may contribute to a less consistently overwhelming caregiving experience while still involving significant caregiving demands. Although most caregivers reported feeling fulfilled and supported in their roles, problems with physical health, mental health, and combining care with daily activities were frequently reported, reflecting the complex and often ambivalent nature of the caregiving experience, where burden and positive appraisal may coexist.

Outside the CarerQol, findings about the level of support families reported further emphasize potential gaps in care. 12% of caregivers reported receiving no help at all, and a few received paid extra help (eg, babysitter or house cleaner). Among those who did receive help, many still reported unmet needs, suggesting that support may be insufficient or poorly targeted. This highlights the importance of ensuring not just the availability of assistance, but its adequacy and appropriateness.

These results also emphasize the critical need for comprehensive assessment and targeted support systems to address caregivers’ physical and psychologic well‐being. This need is further highlighted by a study with 291 caregivers of children with JIA in the United Kingdom, in which 53% of caregivers were unaware of available mental health support services, and 54% reported not being offered mental health support during appointments.^9^

In our statistical models, caregivers’ physical health and receiving extra help scored significantly lower on utility and VAS. In the models including partners’ information, caregivers with partners with mental health conditions also had significantly lower scores, underscoring the importance of caregiver and family health in sustaining caregiving roles. Supporting this, in a study with children with JIA, 82% (n = 218) of caregivers reported that the JIA diagnosis impacted their family's mental health.^9^ We also hypothesize that caregivers experiencing higher caregiving demands are more likely to require extra help, reflecting that caregivers requiring support may already be under a greater burden.

Similarly, lower care‐related quality of life (utility scores) was found among those caring for children using injectable medication. In the models including caregivers’ information only, caregivers’ happiness (CarerQol‐VAS) levels were lower among those caring for children with extra‐articular signs and symptoms or additional health issues beyond JIA. These factors add complexity and greater care demands, as caregivers must manage their own health needs and address multiple health concerns in their children. These findings are consistent with JIA studies and broader pediatric chronic pain literature, in which greater caregiver burden has been associated with poorer child functioning, including greater physical disability, psychosocial difficulties, and school absenteeism.^11^, ^35^, ^36^, ^37^ Together, these findings support the relationship between child disease burden and caregiver quality of life across pediatric chronic conditions.

Interestingly, caregivers of male children reported more caregiving burden (lower utility scores), a finding also observed in another study using a different caregiver burden measure.^8^ This may relate to differences in disease expression or behavior that may affect caregiving demands, although further research is needed to clarify the mechanisms involved.

On the other hand, caring for children aged 10 to 15 years was associated with higher utility scores. In the model that included caregiver information only, higher utility scores were also associated with living with a partner and residing in the Netherlands, possibly reflecting increased family and health care system support. Also, older children typically require less supervision and assistance. Differences in health care system organization between Canada and the Netherlands may have contributed to differences in caregiver quality of life. The Netherlands has a more structured primary care system and broader access to integrated multidisciplinary care, which may facilitate earlier support for families. In contrast, access to allied health and psychosocial services in Canada may be more variable and dependent on the province or insurance coverage. These system‐level differences may influence caregiver support and perceived burden, although this interpretation remains speculative and warrants further investigation. Caregivers of children with inactive disease activity also reported better well‐being (CarerQol‐VAS), highlighting the important link between child health status and caregiver quality of life. Moderate disease activity was also associated with higher CarerQol‐VAS scores compared to high disease activity in the model containing caregiver information only.

These findings have important clinical implications for routine pediatric rheumatology care. Systematic assessment of caregiver well‐being could be incorporated into clinic visits using brief validated tools such as the CarerQol or similar screening measures. Identification of caregivers experiencing high burden could trigger referral pathways to multidisciplinary supports, including social work, psychology, or community‐based services. In addition, providing psychoeducation to families about caregiver stress, coping strategies, and available resources may help normalize caregiver burden and facilitate early help‐seeking. Clinics could also benefit from structured information pathways, including lists of accessible in‐person and online mental health supports. Integrating caregiver‐focused care within routine consultations may improve both caregiver well‐being and family functioning, particularly for those managing higher disease complexity in their child.

Limitations should be considered when interpreting these findings. First, our study may not be fully representative of all caregivers of children with JIA and may have included more caregivers with favorable financial, support, and caregiving circumstances, as caregivers with greater financial stability, stronger social support, and more flexible schedules may have been more able and willing to engage in studies, whereas those experiencing higher psychologic stress or caregiver burden may avoid participating in research. Second, the caregiver sample was highly composed of mothers, which may limit the generalizability of findings to fathers and other caregivers whose experiences may differ. Third, there was a higher percentage of children with oligoarticular JIA. Although this distribution is consistent with the epidemiology of JIA, it may limit the applicability of findings to less common JIA subtypes. Fourth, race and ethnicity were not captured, limiting our ability to examine potential differences in caregiver experiences across diverse populations. Additionally, the effect of adopting the Dutch tariff for the CarerQol‐7D utility score to Canadian caregivers is uncertain. Finally, the regression models were developed using stepwise selection, which has recognized limitations, including potential instability in variable selection. However, multicollinearity among covariates was low (all variance inflation factors ≤3), the initial set of variables included was informed by the literature. Results from the full models are provided in Tables S1 and S2.

As part of the UCAN CAN‐DU and UCAN CURE Consortia, this is the first study to apply the CarerQol instrument to caregivers of children with JIA, a validated questionnaire that captures both positive and negative aspects of caregiving, providing a nuanced understanding of caregiver burden and satisfaction compared to generic tools. By including a large cohort of caregivers across two countries and a broad spectrum of child age, disease stages, treatment, and JIA subtypes, our study offers a novel and relevant contribution. Our results also highlight underexplored dimensions of caregiver burden, such as physical and daily challenges, areas often overlooked in the literature. This emphasizes the need for holistic, family‐centered interventions and support systems that address not only the medical needs of children with JIA but also these broader dimensions of caregiver experience.

This study provides valuable insights into the substantial caregiving challenges faced by parents of children with JIA. Physical and mental burdens, unmet support needs, and child‐ and family‐related factors are key variables associated with lower quality of life. Tailored interventions, focusing on caregiver health, practical support, and family dynamics, are essential for mitigating caregiver burden and promoting the well‐being of both caregivers and their children. Ensuring that caregivers have access to adequate support is crucial for sustaining long‐term caregiving and improving outcomes for families affected by JIA.

## AUTHOR CONTRIBUTIONS

All authors contributed to at least one of the following manuscript preparation roles: conceptualization AND/OR methodology, software, investigation, formal analysis, data curation, visualization, and validation AND drafting or reviewing/editing the final draft. As corresponding author, Dr Currie confirms that all authors have provided the final approval of the version to be published and takes responsibility for the affirmations regarding article submission (eg, not under consideration by another journal), the integrity of the data presented, and the statements regarding compliance with institutional review board/Declaration of Helsinki requirements.

## Supporting information


**Disclosure Form**:

**Figure S1.** Sample selection process, from overall *UCAN CAN‐DU/CURE* data to our study analytic sample.
**Figure S2.** Overall CaerQol‐7D level sum score at baseline. Level sum ranges from 0 (best caregiving situation) to 14 (worst caregiving situation).
**Figure S3.** Caregivers’ perception of help received to care for their children with JIA. Numbers are n (%) indicating the number and proportion of each response.
**Table S1.** Extra help received by caregivers to care for their children with JIA in the previous 3 months.
**Table S2.** Full regression model for CarerQol‐7D utility and CarerQol‐VAS scores, before stepwise selection.
**Table S3.** Stepwise robust regression model for CarerQol‐7D utility scores.
**Table S4.** Stepwise robust regression model for CarerQol‐VAS scores.

## APPENDIX A UCAN CAN‐DU AND UCAN CURE CONSORTIA

Members of the UCAN CAN‐DU and UCAN CURE Consortia are as follows: The Hospital for Sick Children: Rae Yeung, Shirley Tse, Deborah Levy, Ronald Laxer, Brian Feldman, Jennifer Ji Young Lee, and Alisa Rachlis; University of Calgary: Susanne Benseler, Nicole Johnson, Muhammed Dhalla, Paivi Miettunen, Heinrike Schmeling, Marinka Twilt, Rebeka Stevenson, and Deborah Marshall; BC Children's Hospital: Andrea Human, David Cabral, Jaime Guzman, Kim Morishita, Kristin Houghton, Lori Tucker, Tommy Gerschman, Herman Tam, Mercedes Chan, and Ross Petty; Children's Hospital of Eastern Ontario: Roman Jurencak, Nadia Luca, Ciarán Duffy, and Tala El Tal; London Health Sciences Centre: Roberta Berard, Jonathan Park, and Erkan Demirkaya; Children's Hospital Health Centre Winnipeg: Lily Lim; IWK Health Centre: Adam Huber, Bianca Lang, Elizabeth Stringer, Chelsea DeCoste, and Suzanne Ramsey; Stollery Children's Hospital: Dax G. Rumsey, Daniah Basodan, Lillian Lim, Hon Yan Ng, Jeanine McColl, and Tara McGrath; McMaster Children's Hospital: Michelle Batthish, Tania Cellucci, and Liane Heale; Montreal Children's Hospital: Claire LeBlanc, Gaëlle Chédeville, Rosie Scuccimarri, Sarah Campillo, and Piya Lahiry; Janeway Children's/Memorial University: Paul Dancey; Trillium Health Partners: Nickolas Blanchette; University of Saskatchewan: Alan Rosenberg, Mehul Jariwala, Tristan Kerr, and Kate Neufeld; North York General Hospital: Clare Hutchinson and Gordon Soon; Emma Children's Hospital, Amsterdam University Medical Center: Dieneke Schonenberg, Mariken Gruppen, Merlijn van den Berg, and Giske Biesbroek; Sophia Children's Hospital, Erasmus University Medical Center: Philomine van Pelt, Marleen Verkaaik, and Sylvia Kamphuis; Willem‐Alexander Children's Hospital, Leiden University Medical Center: Danielle Brinkman and Petra Hissink Muller; Amalia Children's Hospital, Radboudumc and Sint Maartenskinderkliniek: Ellen Schatorje and Esther Hoppenreijs; Wilhelmina Children's Hospital, University Medical Center Utrecht: Annet van Royen‐Kerkhof, Sebastiaan Vastert, Berent Prakken, Erika Van Nieuwenhove, Joost Swart, Marc Jansen, and Nico Wulffraat; Beatrix Children's Hospital, University Medical Center Groningen: Elizabeth Legger and Wineke Armbrust; Centre for Global e‐health Innovation, University Health Network, University of Toronto: Joseph Cafazzo; Erasmus University and University of Melbourne: Maarten IJzerman; The Hospital for Sick Children: Alexander Mosoiu, Amy Xu, Arthur Cheng, Bruno Pereira, Trang Duong, Harper Cheng, and Brenleigh Jebb; University of Calgary: Gillian R. Currie, Rodrigo Dal Ben, Ravneet Sran, and Elodie Boudes; University of Twente: Michelle Kip; and University Medical Center Utrecht: Sytze de Rook and Regina de Geus.
