# Supplementary material for: The Impact of Caring for a Child With Juvenile Idiopathic Arthritis on Caregiver Quality of Life
Source: ACR Open Rheumatol. 2026 Sep 24;8(10):e90156. doi: 10.1002/acr2.90156 (PMC13612860; doi:10.1002/acr2.90156)
Supplement: Supplementary file 2 [file ACR2-8-e90156-s001.docx]

**Supplementary material**

Figure S1. Sample selection process, from overall *UCAN CAN-DU/CURE* data to our study analytic sample.

Figure S2. Overall CaerQol-7D level sum score at baseline. Level sum ranges from 0 (best caregiving situation) to 14 (worst caregiving situation).

Figure S3. Caregivers’ perception of help received to care for their children with JIA. Numbers are n (%) indicating the number and proportion of each response.

| **Table S1 - Extra help received by caregivers to care for their children with JIA in the previous 3 months*** | | | |
| --- | --- | --- | --- |
| Category | Had help** | How many hours*** | How much (Euro)*** |
| Unpaid extra help | 97 (22%) | 15 (20, 1 – 240) |  |
| Paid extra help (baby-sitter) | 16 (4%) | 18 (28, 3 – 120) | 79 (253, 0 – 2568) |
| Paid extra help (house cleaner) | 16 (4%) | 12 (14, 3 – 52) | 235.5 (246, 0 – 1761) |
| *This data was collected at baseline and refers to the extra help received in the last 3 months prior to that point. Unpaid extra help includes help received from family members, friends, and neighbors. Paid extra help has been adjusted for inflation for 2022 and converted to euro when needed. **N (%), N represents the number of responses, and the percentage indicates the proportion of each response. ***Median (IQR, Min-Max). | | | |

**Table S2 – Full regression model for CarerQol-7D utility and CarerQol-VAS scores, before stepwise selection**

|  | **CarerQol-7D utility**  **F(32, 406) = 2.42, p < 0.001**  Observations: 439  R^2^ / R^2^ adjusted: 0.161 / 0.095 | | | **CarerQol-VAS**  **F(32, 384) = 3.33, p < 0.001**  Observations: 417  R^2^ / R^2^ adjusted: 0.218 / 0.152 | | |
| --- | --- | --- | --- | --- | --- | --- |
| *Predictors* | *Estimates* | *CI* | *p* | *Estimates* | *CI* | *p* |
| (Intercept) | 83.49 | 70.33 – 96.66 | **<0.001** | 8.62 | 6.48 – 10.77 | **<0.001** |
| Caregiver age | -0.11 | -0.31 – 0.09 | 0.289 | -0.01 | -0.04 – 0.02 | 0.522 |
| Number of dependent children | -0.57 | -1.56 – 0.42 | 0.258 | 0.03 | -0.10 – 0.17 | 0.625 |
| Caregiver education  [Technical/trade school] | -1.26 | -3.53 – 1.02 | 0.277 | 0.10 | -0.21 – 0.40 | 0.525 |
| Caregiver education  [High/grade school] | -1.67 | -5.15 – 1.81 | 0.347 | -0.08 | -0.56 – 0.40 | 0.754 |
| Relationship with child [Mother] | -1.04 | -3.98 – 1.90 | 0.488 | -0.30 | -0.69 – 0.10 | 0.139 |
| Receiving an injectable medicine | -2.31 | -4.52 – -0.09 | **0.041** | -0.16 | -0.45 – 0.13 | 0.280 |
| Income level [Below average] | 0.99 | -2.84 – 4.82 | 0.613 | -0.15 | -0.75 – 0.45 | 0.617 |
| Mean household income | 0.00 | -0.00 – 0.00 | 0.317 | -0.00 | -0.00 – 0.00 | 0.348 |
| Country [Netherlands] | 3.92 | 0.67 – 7.17 | **0.018** | -0.43 | -0.95 – 0.09 | 0.108 |
| Child sex [Male] | -2.28 | -4.49 – -0.06 | **0.044** | 0.02 | -0.28 – 0.31 | 0.911 |
| Child age group [5-9] | 1.75 | -1.59 – 5.08 | 0.304 | 0.31 | -0.13 – 0.75 | 0.161 |
| Child age group [10-15] | 4.09 | 0.55 – 7.62 | **0.024** | 0.41 | -0.06 – 0.87 | 0.089 |
| Child age group [16-18] | 3.84 | -0.55 – 8.24 | 0.086 | 0.55 | -0.04 – 1.13 | 0.066 |
| Have comorbidities | -1.15 | -3.61 – 1.31 | 0.360 | -0.32 | -0.65 – 0.01 | 0.060 |
| Have muscular signs and symptoms | -0.20 | -2.46 – 2.07 | 0.865 | -0.25 | -0.55 – 0.05 | 0.109 |
| Have extra-articular signs and symptoms | -0.54 | -3.98 – 2.89 | 0.756 | -0.60 | -1.06 – -0.14 | **0.010** |
| JIA classification  [Extended Oligoarticular JIA] | -5.07 | -10.56 – 0.42 | 0.070 | 0.37 | -0.36 – 1.11 | 0.320 |
| JIA classification  [Oligoarticular JIA (if < 6 months] | -3.36 | -7.30 – 0.58 | 0.094 | 0.08 | -0.44 – 0.60 | 0.764 |
| JIA classification  [Persistent Oligoarticular JIA] | -0.39 | -4.82 – 4.04 | 0.863 | 0.24 | -0.35 – 0.82 | 0.426 |
| JIA classification  [Polyarticular RF negative JIA] | -2.93 | -6.78 – 0.91 | 0.135 | -0.00 | -0.51 – 0.51 | 0.991 |
| JIA classification  [Polyarticular RF positive JIA] | -0.41 | -6.02 – 5.20 | 0.886 | 0.33 | -0.42 – 1.09 | 0.384 |
| JIA classification  [Psoriatic arthritis] | -2.79 | -8.70 – 3.13 | 0.355 | 0.84 | 0.01 – 1.66 | **0.046** |
| JIA classification  [Systemic JIA] | -5.37 | -10.38 – -0.35 | **0.036** | -0.35 | -1.02 – 0.32 | 0.302 |
| JIA classification  [Undifferentiated JIA] | -1.94 | -7.45 – 3.56 | 0.488 | -0.21 | -0.96 – 0.54 | 0.578 |
| Disease status [Inactive] | 0.48 | -4.62 – 5.58 | 0.852 | 0.28 | -0.47 – 1.04 | 0.465 |
| JIA activity state [Inactive] | 0.07 | -5.91 – 6.06 | 0.981 | 0.56 | -0.32 – 1.44 | 0.210 |
| JIA activity state [Minimal] | 1.46 | -2.65 – 5.56 | 0.485 | 0.23 | -0.32 – 0.79 | 0.407 |
| JIA activity state [Moderate] | 1.40 | -1.13 – 3.93 | 0.277 | 0.38 | 0.04 – 0.72 | **0.027** |
| Received extra help | -3.71 | -6.19 – -1.22 | **0.004** | -0.45 | -0.77 – -0.12 | **0.007** |
| Living with a partner | 3.22 | 0.01 – 6.43 | **0.049** | 0.26 | -0.17 – 0.70 | 0.234 |
| Job situation [Unpaid] | 0.76 | -2.32 – 3.84 | 0.627 | 0.20 | -0.22 – 0.63 | 0.349 |
| Physical health condition | -3.57 | -5.58 – -1.56 | **0.001** | -0.54 | -0.81 – -0.28 | **<0.001** |
|  | | | | | | |

**Table S3 – Stepwise robust regression model for CarerQol-7D utility scores**

|  | **Only caregiver information**  Observations: 439 | | | **With spouse/partner information**  Observations: 386 | | |
| --- | --- | --- | --- | --- | --- | --- |
| *Predictors* | *Estimates* | *CI* | *p* | *Estimates* | *CI* | *p* |
| (Intercept) | 79.58 | 76.17 – 82.99 | **<0.001** | 84.42 | 81.85 – 86.98 | **<0.001** |
| Receiving an injectable medicine | -2.17 | -3.87 – -0.46 | **0.013** | -1.74 | -3.58 – 0.10 | 0.063 |
| Country [Netherlands] | 3.38 | 1.73 – 5.04 | **<0.001** | 2.62 | 0.82 – 4.41 | **0.004** |
| Child sex [Male] | -0.83 | -2.57 – 0.92 | 0.354 | -1.15 | -2.99 – 0.70 | 0.223 |
| Child age group [5-9] | 0.90 | -1.73 – 3.53 | 0.503 | 1.29 | -1.48 – 4.05 | 0.362 |
| Child age group [10-15] | 2.75 | 0.41 – 5.10 | **0.022** | 2.10 | -0.33 – 4.54 | 0.090 |
| Child age group [16-18] | 2.25 | -0.61 – 5.11 | 0.122 | 2.40 | -0.63 – 5.44 | 0.121 |
| Received extra help | -3.26 | -5.25 – -1.26 | **0.001** | -2.69 | -4.84 – -0.54 | **0.014** |
| Physical health condition - caregiver | -3.41 | -5.08 – -1.74 | **<0.001** | -2.85 | -4.65 – -1.05 | **0.002** |
| Living with a partner | 3.90 | 1.34 – 6.46 | **0.003** |  |  |  |
| Job situation – unpaid – spouse/partner |  |  |  | -2.24 | -7.10 – 2.62 | 0.366 |
| Physical health condition – spouse/partner |  |  |  | -1.78 | -3.82 – 0.26 | 0.087 |
| Mental health condition – spouse/partner |  |  |  | -11.55 | -16.71 – -6.39 | **<0.001** |
|  |  | | |  |  |  |

**Table S4 – Stepwise robust regression model for CarerQol-VAS scores**

|  | **Only caregiver information**  Observations: 417 | | | **With spouse/partner information**  Observations: 386 | | |
| --- | --- | --- | --- | --- | --- | --- |
| *Predictors* | *Estimates* | *CI* | *p* | *Estimates* | *CI* | *p* |
| (Intercept) | 7.96 | 7.55 – 8.37 | **<0.001** | 8.06 | 7.59 – 8.52 | **<0.001** |
| Country [Netherlands] | -0.32 | -0.59 – -0.05 | **0.019** | -0.37 | -0.67 – -0.07 | **0.015** |
| Child comorbidities | -0.24 | -0.56 – -0.07 | **0.127** | -0.15 | -0.52 – 0.21 | 0.400 |
| Musculoskeletal signs and symptoms | -0.21 | -0.49 – 0.07 | 0.135 | -0.24 | -0.55 – 0.07 | 0.134 |
| Extra-articular signs and symptoms | -0.65 | -1.04 – -0.26 | **0.001** | -0.57 | -1 – -0.15 | **0.008** |
| JIA activity state [Inactive] | 0.93 | 0.44 – 1.41 | **<0.001** | 0.83 | 0.31 – 1.35 | **0.002** |
| JIA activity state [Minimal] | 0.34 | -0.15 – 0.84 | 0.172 | 0.07 | -0.45 – 0.60 | 0.779 |
| JIA activity state [Moderate] | 0.47 | 0.16 – 0.78 | **0.003** | 0.40 | 0.06 – 0.75 | **0.023** |
| Received extra help | -0.52 | -0.81 – -0.22 | **0.001** | -0.41 | -0.74 – -0.08 | **0.016** |
| Physical health condition - caregiver | -0.51 | -0.76 – -0.25 | **<0.001** | -0.44 | -0.73 – -0.16 | **0.002** |
| Job situation – unpaid – spouse/partner |  |  |  | 0 | -0.78 – 0.77 | 0.993 |
| Physical health condition – spouse/partner |  |  |  | -0.25 | -0.58 – 0.07 | 0.126 |
| Mental health condition – spouse/partner |  |  |  | -1.93 | -2.77 – -1.09 | **<0.001** |
